# Viral Mimicry of Interleukin-17A by SARS-CoV-2 ORF8

**DOI:** 10.1128/mbio.00402-22

**Published:** 2022-03-28

**Authors:** Xin Wu, Tian Xia, Woo-Jin Shin, Kwang-Min Yu, Wooram Jung, Alexandra Herrmann, Suan-Sin Foo, Weiqiang Chen, Pengfei Zhang, Jong-Soo Lee, Haryoung Poo, Suzy A. A. Comhair, Lara Jehi, Young Ki Choi, Armin Ensser, Jae U. Jung

**Affiliations:** a Cancer Biology Department, Infection Biology Program, and Global Center for Pathogen and Human Health Research, Lerner Research Institute, Cleveland Clinic, Cleveland, Ohio, USA; b Cleveland Clinic Florida Research & Innovation Center, Port St. Lucie, Florida, USA; c Center for Study of Emerging and Re-emerging Viruses, Korea Virus Research Institute, Institute for Basic Science (IBS), Daejeon, Republic of Korea; d Institute for Clinical and Molecular Virology, University Hospital Erlangen, Friedrich Alexander University Erlangen-Nuremberg, Erlangen, Germany; e College of Veterinary Medicine, Chungnam National University, Daejeon, Republic of Korea; f Infectious Disease Research Center, Korea Research Institute of Bioscience and Biotechnology, Daejeon, Republic of Korea; g Respiratory Institute, Lerner Research Institute, Cleveland Clinic, Cleveland, Ohio, USA; h Department of Neurology, Epilepsy Center, Cleveland Clinic, Cleveland, Ohio, USA; University of North Carolina, Chapel Hill

**Keywords:** COVID-19, viral IL-17, inflammation

## Abstract

Severe acute respiratory syndrome coronavirus 2 (SARS-CoV-2) infection triggers cytokine-mediated inflammation, leading to a myriad of clinical presentations in COVID-19. The SARS-CoV-2 open reading frame 8 (ORF8) is a secreted and rapidly evolving glycoprotein. Patients infected with SARS-CoV-2 variants with ORF8 deleted are associated with mild disease outcomes, but the molecular mechanism behind this is unknown. Here, we report that SARS-CoV-2 ORF8 is a viral cytokine that is similar to but distinct from interleukin 17A (IL-17A) as it induces stronger and broader human IL-17 receptor (hIL-17R) signaling than IL-17A. ORF8 primarily targeted blood monocytes and induced the heterodimerization of hIL-17RA and hIL-17RC, triggering a robust inflammatory response. Transcriptome analysis revealed that besides its activation of the hIL-17R pathway, ORF8 upregulated gene expression for fibrosis signaling and coagulation dysregulation. A naturally occurring ORF8 L84S variant that was highly associated with mild COVID-19 showed reduced hIL-17RA binding and attenuated inflammatory responses. This study reveals how SARS-CoV-2 ORF8 by a viral mimicry of the IL-17 cytokine contributes to COVID-19 severe inflammation.

## INTRODUCTION

The Coronavirus Disease 2019 (COVID-19) pandemic triggered by the severe acute respiratory syndrome coronavirus 2 (SARS-CoV-2) is a major health crisis that has affected more than 200 million individuals and caused over 4 million deaths worldwide (1, 2). Patients with severe COVID-19 infection show signs of acute respiratory distress syndrome (ARDS) and multiple organ failure induced by cytokine storm, which is a life-threatening condition broadly characterized by the overproduction of inflammatory cytokines and hyperactivation of immune cells (3, 4). Interleukin 17A (IL-17A) is a 155-amino-acid (aa), secreted glycosylated cytokine that plays a crucial role in triggering cytokine storms and is associated with alveolar inflammation and the destruction of lung parenchyma in ARDS (5, 6). In addition, increased levels of IL-17A have been detected in SARS-CoV-, Middle East respiratory syndrome (MERS-CoV)-, and SARS-CoV-2-infected patients, especially in those with severe symptoms, suggesting a crucial role of IL-17 signaling in exacerbating severe ARDS (6–8). In fact, SARS-CoV-2 infection induced stronger activation of IL-17 signaling than other respiratory viruses. Therefore, aberrant activation of IL-17 signaling-triggered lung inflammation and ARDS may lead to the onset of severe COVID-19 (9).

The IL-17 cytokine family consists of six members (IL-17A to IL-17F) and signals through the IL-17 receptor family, including IL-17RA to IL-17RE. Functional IL-17R is a complex that typically consists of IL-17RA, the founding receptor of IL-17R family, and one other receptor subunit to form the heterodimer, thus allowing the interaction with various IL-17 cytokines (10–15). For instance, IL-17RA dimerizes with IL-17RC to transduce signaling in response to IL-17A. This receptor dimerization triggers the downstream activation of several transcription factors such as NF-κB, resulting in the production of inflammatory cytokines/chemokines and contributing to the elimination of microorganisms’ infection (16, 17). However, aberrant activation of the IL-17 receptors leads to the development of a cytokine storm, resulting in inflammatory diseases such as psoriasis, rheumatoid arthritis, and ARDS (18). Targeting of IL-17RA signaling has been adopted as a strategy to treat autoimmune diseases such as psoriasis (19). Interestingly, psoriasis patients who had been previously treated with IL-17A antagonists were associated with relatively mild or even asymptomatic COVID-19, suggesting a protective role of IL-17 inhibitors in ARDS (20).

The SARS-CoV-2 genome encodes a total of 29 proteins. Four form the viral structure and 16 nonstructural proteins (Nsp) processed by 3C-like protease, Nsp5, as well as a papain-like protease, Nsp3. The virus also encodes nine accessory proteins, which are not required for viral replication but essential in hijacking the host innate immune system for efficient infection (2, 21). Interestingly, patients infected with a SARS-CoV-2 variant carrying the Δ382 deletion or the L84S mutation of the open reading frame 8 (ORF8) accessory protein experienced milder inflammatory responses and attenuated disease outcomes, suggesting a crucial role for ORF8 in triggering COVID-19 proinflammatory sequelae (22, 23). The SARS-CoV-2 ORF8 consists of 121 amino acids, including an N-terminal signal peptide (1 to 15 aa) and a C-terminal immunoglobulin (Ig)-like domain (16 to 121 aa). It has been identified as a secreted, highly immunogenic, and rapidly evolving protein (24–26). Sequence alignment shows that the SARS-CoV-2 ORF8 shares high similarity with Bat-RaTG13-CoV ORF8 (95%) but low similarity with SARS-CoV ORF8 (30%), suggesting that SARS-CoV-2 ORF8 may have originated from the Bat-RaTG13-CoV ORF8 (27). These features of a secreted Ig-like family protein suggest the potential immunomodulatory role of ORF8 in virus-induced inflammation.

Here, we report that secreted SARS-CoV-2 ORF8 specifically binds to human IL-17RA (hIL-17RA) and hIL-17RC on the surface of human blood monocytes. ORF8 interaction with hIL-17RA/C induced a markedly stronger inflammatory response than hIL-17A. We also demonstrated that the disruption of ORF8 and hIL-17R interaction attenuated its proinflammatory responses and that a variant form of ORF8 associated with mild COVID-19 (L84S) showed decreased binding activity to hIL-17RA, resulting in an attenuated proinflammatory response (23, 28). Intriguingly, transcriptomic analysis revealed that besides its activation of the hIL-17R pathway, ORF8 uniquely increased expression of key factors for fibrosis and coagulation dysregulation. Overall, our findings uncover SARS-CoV-2 ORF8 as a viral cytokine that is similar but distinct from hILR-17A, suggesting a potential therapeutic target for COVID-19.

## RESULTS

### Secreted SARS-CoV-2 ORF8 primarily binds to CD14^+^ blood monocytes.

**FIG 1:**
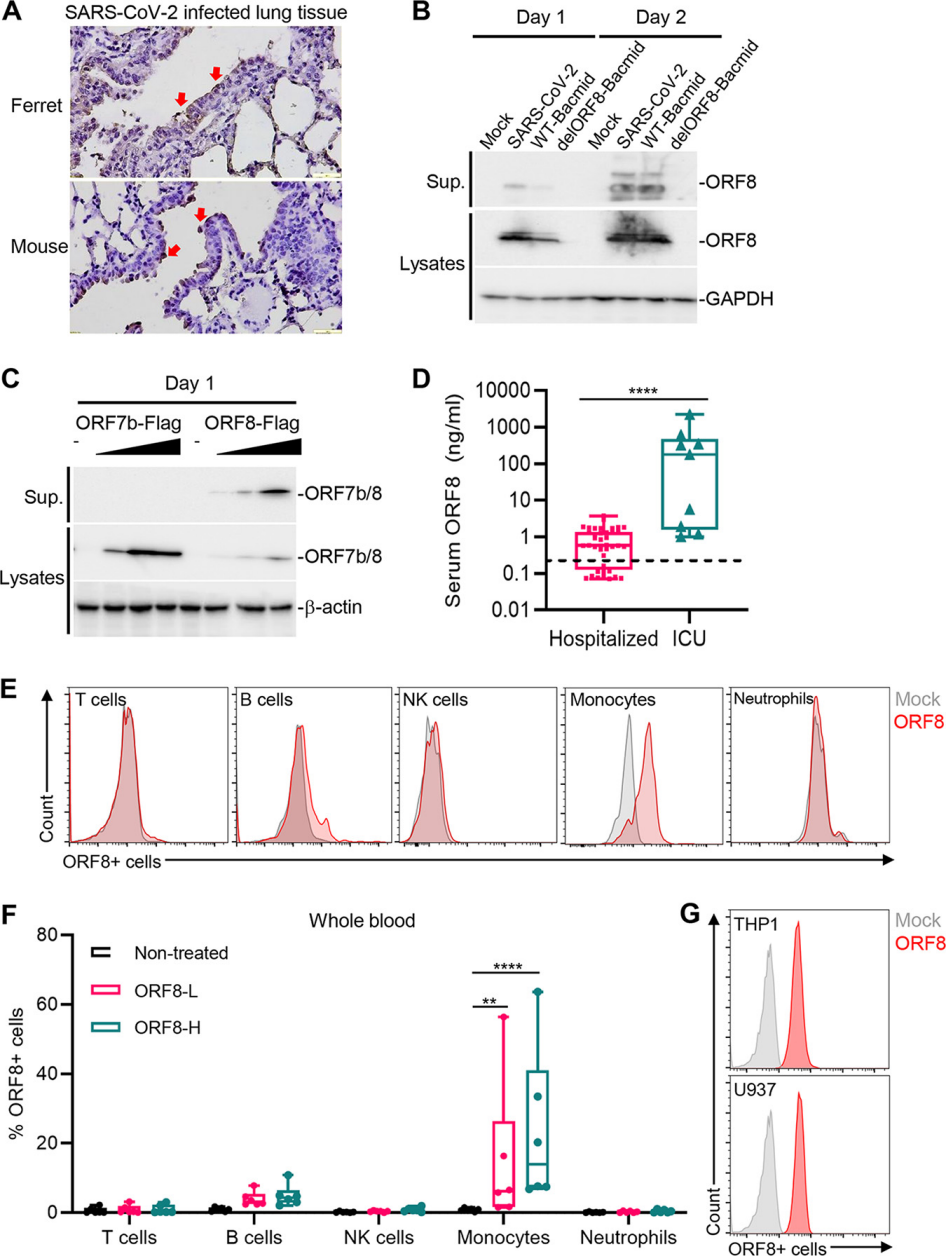
Secreted ORF8 binds primarily to CD14^+^ blood monocytes. (A) ORF8 is highly expressed in the lung tissues of SARS-CoV-2-infected ferrets and mice. Immunohistochemistry showed ORF8 expression after SARS-CoV-2 infection in lung sections of ferrets and mice. Scale bar, 1 mm. Arrowheads mark ORF8 expression. (B) ORF8 is detected in the supernatants of SARS-CoV-2-infected Vero cells. At 24 h or 48 h postinfection, cell lysates and culture supernatants (Sup.) were harvested, and ORF8 protein was detected by immunoblotting using ORF8 antibody. (C) ORF8 is secreted into the supernatants of transfected 293T cells. Plasmid DNA encoding the SARS-CoV-2 ORF7b or ORF8 with a Flag tag was transfected into 293T cells. At 48 h posttransfection with SARS-CoV-2 ORF7b-Flag or ORF8-Flag, cell lysates and supernatants (Sup.) were harvested, and ORF7b/ORF8 proteins were detected by immunoblotting using Flag antibody. (D) SARS-CoV-2 ORF8 concentrations (ng/mL) in the sera of hospitalized COVID-19 patients (*n* = 40) and ICU-admitted COVID-19 patients (*n *= 9). The horizontal line indicates the median value of the pre-COVID-19 pandemic sera of healthy subjects (*n* = 9). (E, F) Purified Flag-tagged ORF8 protein primarily binds to monocytes in human blood cells. Whole blood derived from healthy donors (*n *= 6) was incubated with purified ORF8-Flag for 2 h. Subsequently, granulocytes and PBMCs were isolated and subjected to flow cytometry with Flag antibody. Various immune cells were gated by specific antibodies: CD45^+^ CD3^+^ T cells, CD45^+^ CD19^+^ B cells, CD45^+^ CD14^+^ monocytes, CD45^+^ CD56^+^ NK cells, and CD45^+^ CD16^+^ CD11b^+^ neutrophils. (G) Purified ORF8-Flag binds to monocytic cell lines. THP-1 and U937 cells were incubated with purified ORF8-Flag for 2 h, and then ORF8-Flag binding activity was analyzed by flow cytometry. *P* values were calculated by Mann-Whitney *U* test (D) and two-way ANOVA with Dunnett test for multiple comparisons (F). *, *P* < 0.05; **, *P* < 0.01; ***, *P* < 0.005; ****, *P* < 0.0001.

Previous studies have shown that antibodies and peptides against ORF8 are readily detected in the sera of COVID-19 patients (25, 26), suggesting that ORF8 is a secreted viral protein with highly immunogenic properties (29). We found that ORF8 was readily detected in lung bronchial epithelial cells of SARS-CoV-2-infected ferrets and mice (Fig. 1A) and in the supernatants of Vero cells infected with wild-type (WT) virus but not virus variants with ORF8 deleted (Fig. 1B). Since ORF7b is also predicted to be a membrane protein with an N-terminal signal peptide, ORF7b was included as a control. This showed that ORF8, not ORF7b, was detected in the supernatants of transfected 293T cells (Fig. 1C), demonstrating the secretory nature of ORF8. Serum samples from hospitalized COVID-19 patients (*n = *40), intensive care unit (ICU)-admitted COVID-19 patients (*n = *9), and healthy individuals pre-COVID-19 pandemic (*n =* 9) were subjected to ORF8 enzyme-linked immunosorbent assay (ELISA) to determine the amount of circulating ORF8. This showed that the median serum levels of ORF8 were 1 ng/mL in hospitalized COVID-19 patients and 414.5 ng/mL in ICU-admitted COVID-19 patients (Fig. 1D). These data indicate that SARS-CoV-2 ORF8 is a secreted protein, and its amount in serum is strikingly high in ICU-admitted COVID-19 patients.

To determine whether secreted ORF8 targeted specific blood immune cells, we incubated human whole blood obtained from healthy donors (*n *= 6) with glycosylated ORF8-Flag purified from 293T cell supernatants (see Fig. S1A in the supplemental material). At 2 h postincubation, CD45^+^ immune cells from whole-blood specimens were gated into five major blood immune subsets (T cells, B cells, NK cells, monocytes, and neutrophils), followed by anti-Flag antibody staining to determine ORF8-interacting cells (Fig. S1B). This identified CD14^+^ monocytes as primary target cells for ORF8 binding (Fig. 1E and F). Furthermore, ORF8-Flag strongly bound to cells of the human monocytic cell lines THP-1 and U937 (Fig. 1G). These results indicate that monocytes are the primary target cells for ORF8 binding.

10.1128/mbio.00402-22.1 FIG S1 Secreted ORF8 primarily binds to CD14^+^ blood monocytes. (A) SDS-PAGE image showing InstantBlue-stained Flag-tagged recombinant protein fractions that were purified with Flag M2 beads from concentrated supernatants (ORF8) or cell lysates (ORF7b) of transfected 293T cells. (B) Gating strategy for each immune subset within human blood. Neutrophils and PBMCs were isolated from whole blood of healthy donors for FACS analysis. Major blood immune subsets, including CD3^+^ CD19^−^ T cells, CD3^−^ CD19^+^ B cells, CD14^−^ CD56^+^ NK cells, CD14^+^ CD56^−^ monocytes, and CD16^+^ CD11b^+^ cells, were gated from the CD45^+^ population. The gating of ORF8^+^ cells was based on the total population of gated monocytes, NK cells, T cells, B cells, or neutrophils. Download FIG S1, PDF file, 0.4 MB. Copyright © 2022 Wu et al. 2022 Wu et al. https://creativecommons.org/licenses/by/4.0/ This content is distributed under the terms of the Creative Commons Attribution 4.0 International license.

### ORF8 interacts with IL-17RA to activate inflammatory responses.

**FIG 2:**
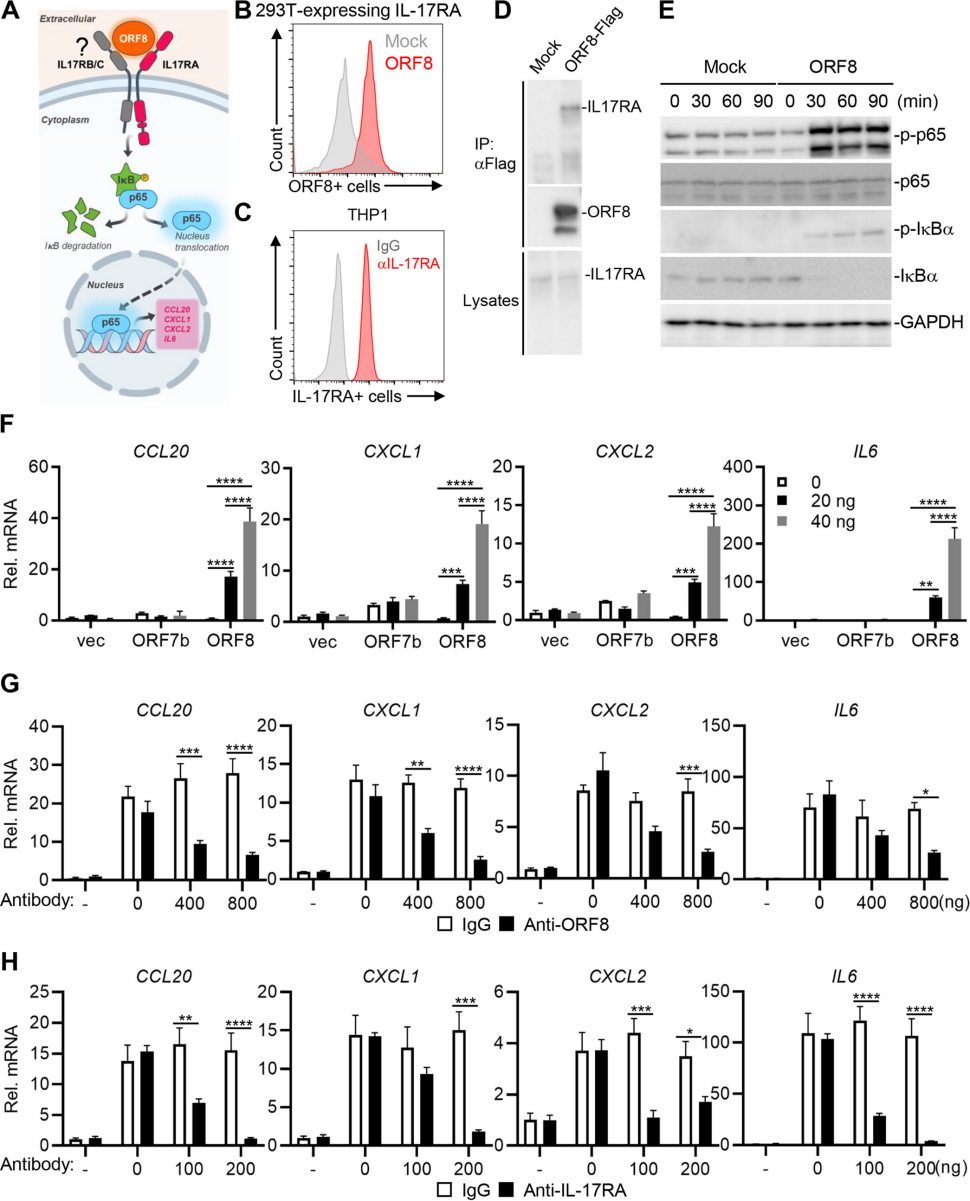
ORF8 interacts with hIL-17RA to activate inflammatory responses. (A) Hypothesis model of ORF8-mediated IL-17RA signaling. (B) Purified ORF8-Flag interacts with 293T-expressing hIL-17RA. At 24 h posttransfection with vector or hIL-17RA expression vector, 293T cells were subsequently incubated for 30 min with Flag-tagged ORF8 or untreated, followed by flow cytometry with anti-Flag antibody. (C) THP-1 cells express high levels of surface hIL-17RA. THP-1 cells were stained with hIL-17RA antibody and followed by flow cytometry. (D) Purified ORF8-Flag protein interacts with endogenous IL-17RA in THP-1 cells. THP-1 cells were incubated with purified ORF8-Flag proteins for 2 h, and cell lysates were immunoprecipitated with anti-Flag, followed by immunoblotting with indicated antibodies. (E) ORF8 induces the phosphorylation of p65 and IκΒα. THP-1 cells were treated with or without ORF8 (50 ng/mL) for the indicated times, followed by immunoblots with the indicated antibodies. (F) ORF8 treatment increases inflammatory cytokine gene expression. THP-1 cells were treated with ORF7b or ORF8 at the indicated concentrations for 16 h, followed by RT-qPCR. (G) ORF8 neutralizing antibody blocks ORF8 function. THP-1 cells were treated with purified ORF8 (20 ng/mL) and its antibody at the indicated concentrations for 16 h, followed by RT-qPCR. (H) IL-17RA neutralizing antibody blocks ORF8 function. THP-1 cells were treated with control IgG or IL-17RA neutralizing antibody at the indicated concentrations for 2 h and then stimulated with ORF8 for 16 h, followed by RT-qPCR. *P* values were calculated by two-way ANOVA with Tukey’s test in panel F or Bonferroni’s test in panels G and H for multiple comparisons. *, *P* < 0.05; **, *P* < 0.01; ***, *P* < 0.005; ****, *P* < 0.0001.

Previous proteomic studies have reported that ORF8 interacts with hIL-17RA, an essential receptor for IL-17 signaling, which forms a heterodimer with hIL-17RB or hIL-17RC to transduce signaling in the activation of proinflammatory responses (13, 30, 31) (Fig. 2A). Consistently, purified ORF8-Flag strongly bound to 293T cells overexpressing hIL-17RA (Fig. 2B). As THP-1 cells expressed a high level of surface hIL-17RA (Fig. 2C), THP-1 cells were incubated with purified ORF8-Flag, followed by immunoprecipitation and immunoblotting. This showed that purified ORF8-Flag readily interacted with endogenous hIL-17RA in THP-1 cells (Fig. 2D). Upon ORF8 stimulation, the phosphorylation of p65 and IκΒα as well as the degradation of IκΒα were markedly increased in ORF8-treated THP-1 cells compared with mock-treated THP-1 cells (Fig. 2E). In addition, treatment of THP-1 or U937 cells with purified ORF8, but not with purified ORF7b, induced expression of genes coding for proinflammatory factors of the IL-17 receptor pathway, including *CCL20*, *CXCL1*, *CXCL2*, and *IL6*, in a dose-dependent manner (Fig. 2F; see Fig. S2A in the supplemental material) (32). Conversely, treatment of anti-ORF8 or anti-hIL-17RA antibody considerably attenuated ORF8-induced *CCL20*, *CXCL1*, CXCL2, and *IL6* expression in a dose-dependent manner, indicating that the ORF8–IL-17RA interaction is critical for the induction of proinflammatory cytokine gene expression (Fig. 2G and H).

10.1128/mbio.00402-22.2 FIG S2 ORF8 specifically interacts with hIL-17RA. (A) ORF8 increases the mRNA level of inflammatory genes in U937 cells. U937 cells were treated with ORF8 at the indicated concentration or left untreated for 16 h. The mRNA levels of the indicated genes in the cells were measured by qPCR experiments. (B) Purified ORF8-Flag protein showed no binding activity to mouse IL-17RA. Mouse IL-17RA expression plasmids or empty vector was transfected into 293T cells. At 24 h posttransfection, 293T cells were subsequently incubated for 30 min with Flag-tagged ORF8 or untreated, followed by staining with anti-Flag antibody for flow cytometry. (C) Purified ORF8 protein showed no binding activity to RAW264.7 cells. RAW264.7 cells were incubated with purified ORF8-Flag protein or mIL-17A for 2 h, and then the binding activity was analyzed by flow cytometry. (D, E) ORF8 is unable to increase the mRNA level of inflammatory genes in RAW264.7 cells or BMDMs. RAW264.7 cells (D) or BMDMs (E) were treated with mIL-17A or ORF8 at the concentration indicated or left untreated for 16 h. The mRNA levels of the indicated genes in the cells were measured by qPCR experiments. *P* values were calculated by two-way analysis of variance (ANOVA) with Dunnett’s test in panel A and one-way ANOVA with Tukey’s posttest in panels D and E. *, *P* < 0.05; **, *P* < 0.01; ***, *P* < 0.005; ****, *P* < 0.0001. Download FIG S2, PDF file, 0.3 MB. Copyright © 2022 Wu et al. 2022 Wu et al. https://creativecommons.org/licenses/by/4.0/ This content is distributed under the terms of the Creative Commons Attribution 4.0 International license.

It should be noted that a previous study has shown that bacterially purified ORF8 interaction with IL-17RA induces the production of proinflammatory factors in the mouse monocyte/macrophage RAW264.7 cell line and in the mouse model (33). To determine if there is species specificity for the activation of ORF8/IL-17 receptor signaling, we treated 293T cells overexpressing mouse IL-17RA (mIL-17RA) or mouse RAW264.7 cells with glycosylated ORF8 purified from 293T cells and compared their interaction and proinflammatory induction capability with those of mIL-17A. Surprisingly, ORF8 did not show detectable binding to surface-expressed mIL-17RA on 293T cells (Fig. S2B). High surface binding activity of mIL-17A was observed on RAW264.7 cells, but little or no binding activity of purified ORF8 was detected under the same conditions (Fig. S2C). Consistent with the lack of its mIL-17RA binding activity, ORF8 treatment did not induced expression of mouse *Ccl20* and *Cxcl1* in RAW264.7 and bone marrow-derived macrophages (BMDMs), whereas mIL-17A treatment apparently induced expression of mouse *Ccl20* and *Cxcl1* in RAW264.7 cells (Fig. S2D and E). Collectively, these results suggest that ORF8 specifically interacts with hIL-17RA, activating downstream NF-κB signaling and triggering proinflammation.

### ORF8 is a potent viral mimic of hIL-17A.

**FIG 3:**
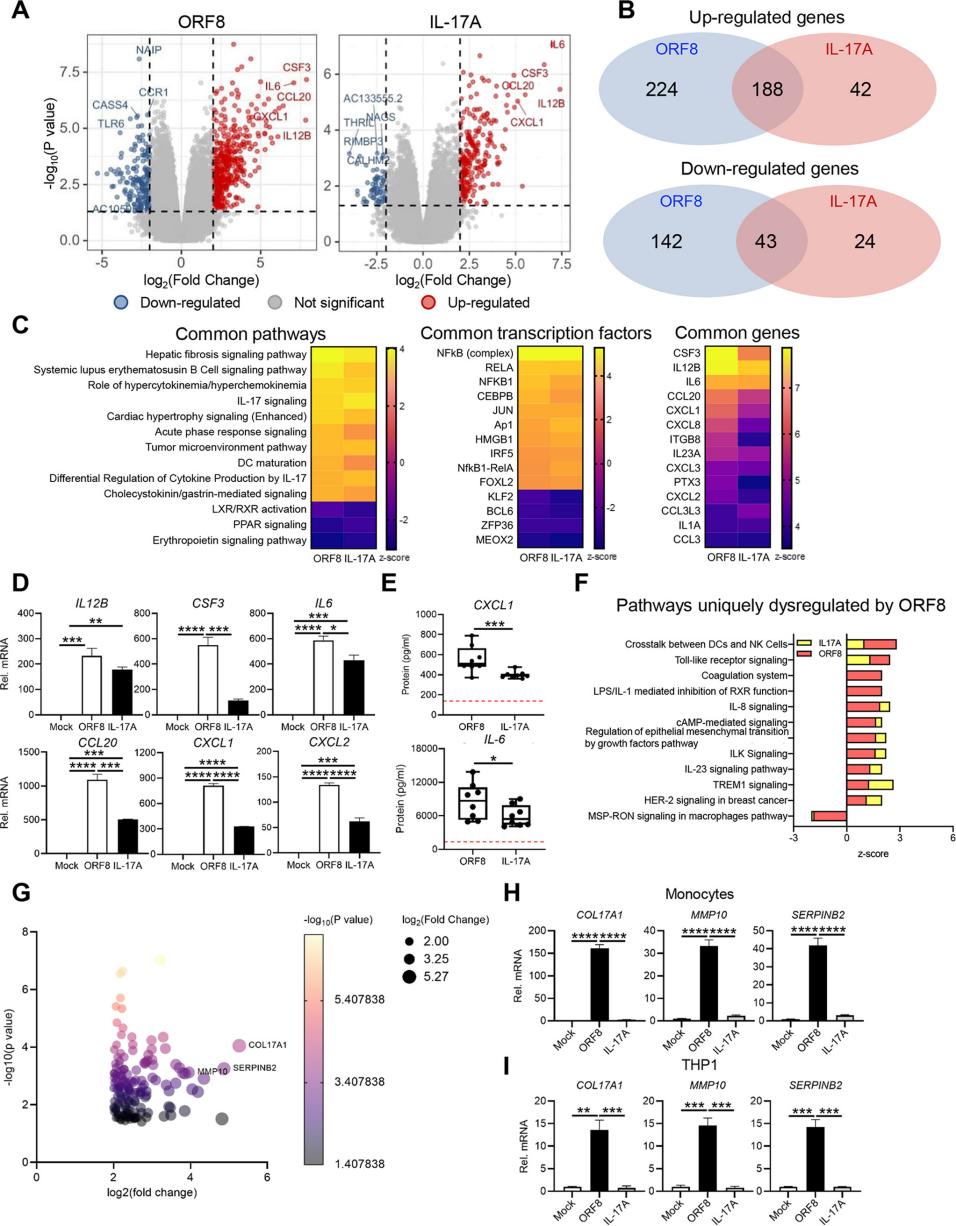
ORF8 is a potent viral mimic of hIL-17A. After 2 h of mock treatment or treatment with ORF8 or hIL-17A, human monocytes and plasma were isolated from whole blood of male and female donors (*n* = 4), and their RNAs were subjected to RNA-Seq analysis. (A) Volcano plot analysis of differential gene expressions in ORF8 and hIL-17A treatment compared to mock treatment (*n* = 4). (B) Comparison of significantly increased or decreased gene expression between monocytes treated with ORF8 or hIL-17A is presented as a Venn diagram. (C) Comparison analysis of IPA canonical pathways significantly enriched in the ORF8- or hIL-17A-treated group compared to the control group (left panel). A heat map of the top 10 transcription factors commonly up- or downregulated by ORF8 and hIL-17A is shown in the middle panel, and a heat map of NF-κB-regulated target genes commonly affected by ORF8 or hIL-17A treatment is shown in the right panel. A negative Z score means the inactivation of genes or pathways compared to the control group, and a positive Z score means the activation of genes or pathways compared to the control group. (D) NF-κB-regulated genes (*IL12B*, CSF3, *IL6*, *CCL20*, *CXCL1*, and *CXCL2*) were normalized to GAPDH and expressed as fold change relative to mock controls. (E) CXCL1 and IL-6 ELISA of plasma of ORF8- or hIL-17A-treated whole blood, with horizontal lines indicating median values of mock-treated whole blood observed. (F) IPA of uniquely ORF8-regulated pathways. (G) Immune profiling of ORF8-upregulated unique genes. (H) RT-qPCR analysis of ORF8-upregulated unique genes (*COL17A1*, *MMP10*, and *SERPINB2*) in primary monocytes that were normalized to GAPDH and expressed as fold change relative to mock controls. (I) RT-qPCR analysis of ORF8-upregulated unique genes (*COL17A1*, *MMP10*, and *SERPINB2*) in THP-1 cells were normalized to GAPDH and expressed as fold change relative to mock controls. *P* values were calculated by one-way ANOVA with Tukey’s posttest in panels D, H, and I and multiple *t* test in panel E. *, *P* < 0.05; **, *P* < 0.01; ***, *P* < 0.005; ****, *P* < 0.0001.

Monocytes isolated from whole blood of healthy donors were treated with either ORF8 or hIL-17A, and total RNAs were extracted for RNA-Seq analysis (see Fig. S3A and B in the supplemental material). The majority of upregulated host genes (*CCL20*, *CXCL1*, *IL6*) were similar between ORF8-treated monocytes and hIL-17A-treated monocytes (Fig. 3A). Transcription of totals of 412 and 230 genes were upregulated in ORF8-treated and hIL-17A-treated monocytes, respectively, compared to mock-treated monocytes, and 224 and 42 genes were downregulated in ORF8-treated and hIL17A-treated monocytes, respectively (Fig. 3B). Among these upregulated genes, 188 genes (81.7%) were shared between hIL17A- and ORF8-treated monocytes. Among the downregulated genes, 43 (64%) genes were shared between hIL17A- and ORF8-treated monocytes (Fig. 3B). Figure 3C shows pathways, transcription factors, and specific genes from Ingenuity Pathway Analysis (IPA) that were commonly induced in ORF8- or hIL17A-treated monocytes (Fig. 3C). Specifically, *IL12B*, *CSF3*, *IL6*, *CCL20*, *CXCL1*, and *CXCL2* were commonly upregulated, but their levels of upregulation were significantly higher in ORF8-treated monocytes than in hIL17A-treated monocytes (Fig. 3D). In addition, ELISA confirmed that CXCL1 and IL-6 levels were significantly higher in the plasma of ORF8-treated blood than that of hIL-17A-treated blood (Fig. 3E). While expression of many genes was commonly induced by ORF8 and hIL-17A, a number of pathways and gene transcription activities, including *COL17A1*, *MMP10*, and *SERPINB2*, were induced only in ORF8-treated primary monocytes and THP-1 cells, but not in hIL-17A-treated cells (Fig. 3F to I). These data indicate that ORF8 induces intracellular signaling pathways similar but also distinct from hIL-17A.

10.1128/mbio.00402-22.3 FIG S3 ORF8 is a potent viral mimic of hIL-17A. (A) Efficiency of monocyte isolation from PBMCs. (B) Principal-component analysis result. The figure displays a three-dimensional scatter plot of the first three principal components (PCs) of the data. Each point represents an RNA-Seq sample. Samples with similar gene expression profiles are clustered together. Sample groups are indicated by using different colors as indicated in the legend provided. Download FIG S3, PDF file, 0.2 MB. Copyright © 2022 Wu et al. 2022 Wu et al. https://creativecommons.org/licenses/by/4.0/ This content is distributed under the terms of the Creative Commons Attribution 4.0 International license.

### ORF8 binding enhances the heterodimerization of hIL-17RA and hIL-17RC.

**FIG 4:**
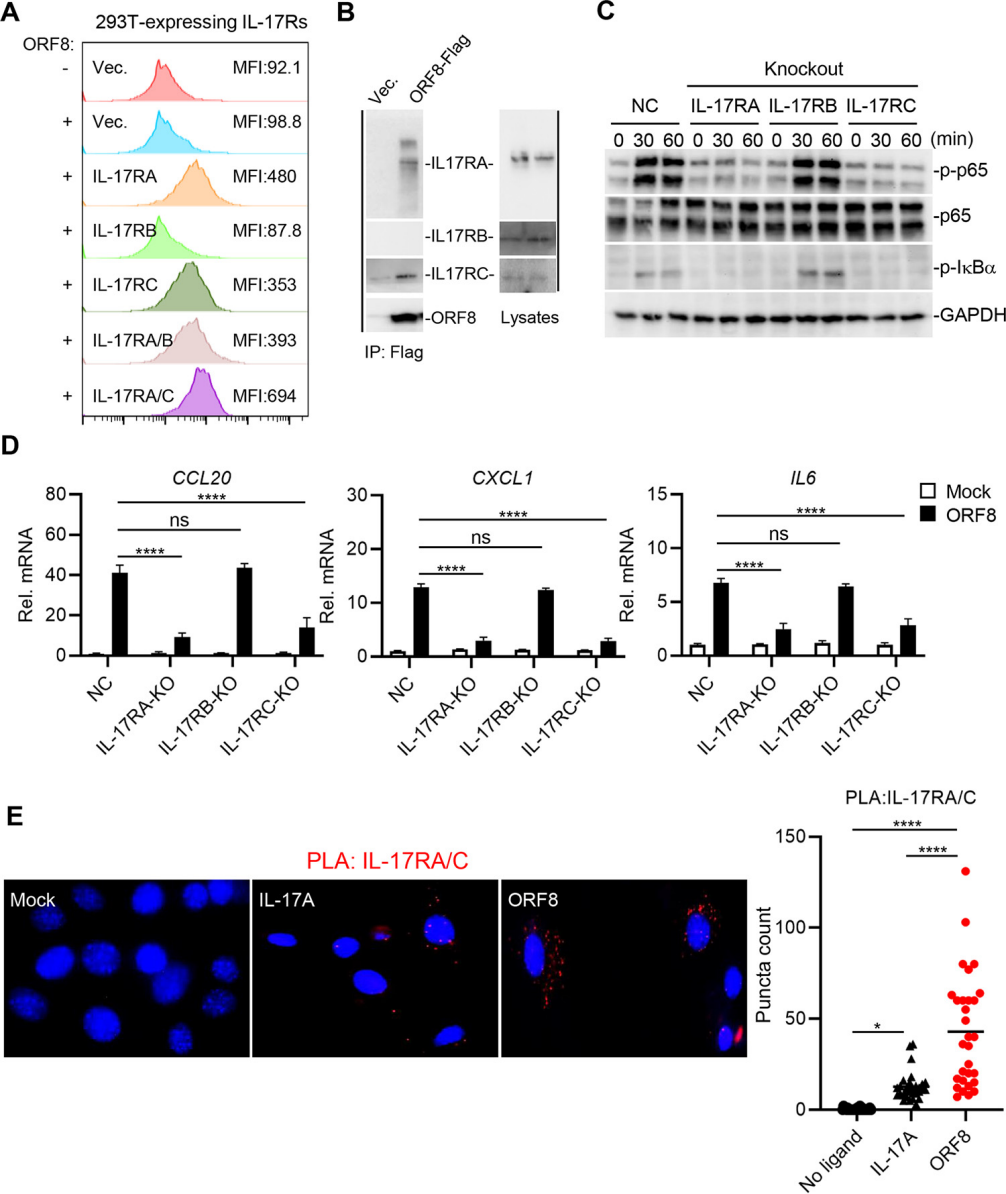
ORF8 binding enhances the heterodimerization of hIL-17RA/C. (A) Purified ORF8-Flag interacts with hIL-17RA and hIL-17RC. At 24 h posttransfection of the IL-17 receptor construct, 293T cells were subsequently incubated for 30 min with mock-treated or purified ORF8-Flag, followed by staining with anti-Flag antibody for flow cytometry. (B) Purified ORF8-Flag protein interacts with endogenous IL-17RA and IL-17RC in THP-1 cells. THP-1 cells were incubated with purified ORF8-Flag for 2 h. Cell lysates were immunoprecipitated with anti-Flag, followed by immunoblotting with the indicated antibodies. (C) Effects of IL-17RA/B/C deficiency on the ORF8-induced phosphorylation of p65 and IκBα. IL-17RA, IL-17RB, or IL-17RC knockout (KO) THP-1 cells were generated by the CRISPR-Cas9 method. IL-17RA KO, IL-17RB KO, IL-17RC KO, or control THP-1 cells were mock treated or treated with ORF8 for the indicated times, followed by immunoblotting with the indicated antibodies. (D) Effects of IL-17RA/B/C deficiency on ORF8-mediated cytokine gene expression. IL-17RA KO, IL-17RB KO, IL-17RC KO, or control THP-1 cells were mock treated or treated with ORF8 for 16 h, followed by qRT-PCR to measure the mRNA levels of indicated genes. (E) Proximity ligation assay (PLA) of IL-17RA/C heterodimerization in NIH 3T3 cells. At 24 h posttransfection with HA-hIL-17RA and Myc-hIL-17RC, NIH 3T3 cells were treated with hIL-17A or purified ORF8 for 30 min, followed by PLA. *P* values were calculated by two-way ANOVA with Dunnett’s posttest in panel D or one-way ANOVA with Tukey’s posttest in panel E. *, *P* < 0.05; **, *P* < 0.01; ***, *P* < 0.005; ****, *P* < 0.0001.

IL-17RA usually forms a heterodimer with IL-17RB or IL-17RC to transduce signaling in the activation of inflammatory responses (11, 12). To determine if ORF8 could bind alternative hIL-17 receptors, we tested the interaction of purified ORF8 with hIL-17RA-, hIL-17RB-, and/or hIL-17RC-expressing 293T cells. hIL-17RA- and hIL-17RC-coexpressing cells showed the highest binding activity to ORF8, followed by hIL-17RA-expressing cells, and then hIL-17RC-expressing cells (Fig. 4A). In contrast, hIL-17RB-expressing cells showed no binding activity to ORF8 (Fig. 4A). Furthermore, endogenous hIL-17RA and hIL-17RC, but not hIL-17RB, interacted with ORF8 in THP-1 cells (Fig. 4B).

To further investigate if the ORF8 interaction with hIL-17RA/C complex affects downstream signaling, we generated individual hIL-17RA-, hIL-17RB- or hIL-17RC-knockout (KO) THP-1 or U937 cells by a CRISPR-single guide RNA (sgRNA) approach (see Fig. S4A in the supplemental material). Upon ORF8 treatment, the phosphorylation levels of p65 and IκBα, which indicate the activation of NF-κB pathways, were markedly decreased in hIL-17RA- or hIL-17RC-deficient cells, but not in hIL-17RB-deficient cells, compared to parental THP-1 cells (Fig. 4C). Furthermore, the deficiency of hIL-17RA or hIL-17RC, but not hIL-17RB, in THP-1 and U937 cells resulted in the considerable attenuation of ORF8-induced *CCL10*, *CXCL1*, and *IL6* expression (Fig. 4D; Fig. S4B). These results strongly indicate that ORF8 interacts with hIL-17RA and hIL-17RC, inducing downstream signaling.

10.1128/mbio.00402-22.4 FIG S4 ORF8 mediates dimerization of IL-17RA/C. (A) Knockout (KO) efficiency of IL-17RA KO, IL-17RB KO, and IL-17RC in THP-1 cells was analyzed by immunoblots with the indicated antibodies. (B) Effects of IL-17RA/B/C deficiency on ORF8-mediated cytokine gene expression. IL-17RA KO, IL-17RB KO, IL-17RC KO, or control U937 cells were mock treated or treated with ORF8 for 16 h, followed by RT-qPCR to measure the mRNA levels of indicated genes. *P* values were calculated by two-way ANOVA with Bonferroni’s posttest in panel C. *, *P* < 0.05; **, *P* < 0.01; ***, *P* < 0.005; ****, *P* < 0.0001. Download FIG S4, PDF file, 0.2 MB. Copyright © 2022 Wu et al. 2022 Wu et al. https://creativecommons.org/licenses/by/4.0/ This content is distributed under the terms of the Creative Commons Attribution 4.0 International license.

Since the heterodimerization of IL-17RA and IL-17RC (IL-17RA/C) is a critical step of IL-17 signaling activation, NIH 3T3 cells expressing hemagglutinin (HA)-tagged hIL-17RA and Myc-tagged hIL-17RC were treated with purified ORF8 or hIL-17A, and then IL-17RA/C heterodimerization was measured by proximity ligation assay (PLA). This showed that the heterodimerization of hIL-17RA/C was markedly increased in ORF8-treated cells compared to that in hIL-17A-treated cells (Fig. 4E), indicating that ORF8 binding enhances the heterodimerization of hIL-17RA/C, thereby activating efficient inflammatory responses.

### ORF8 mutational analysis for hIL-17RA and hIL-17RC interaction.

**FIG 5:**
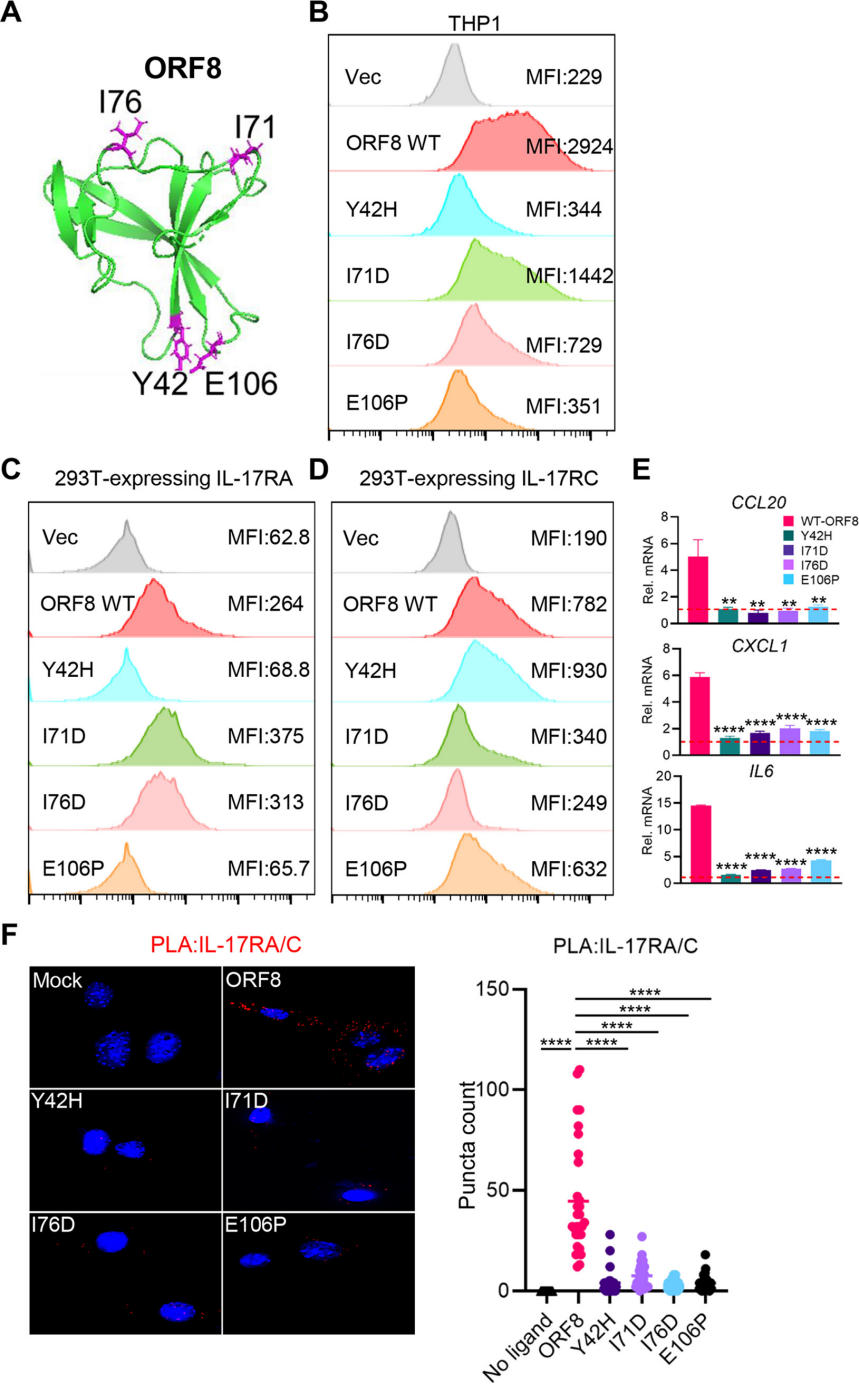
ORF8 mutational analysis for hIL-17RA and hIL-17RC interaction. (A) Y42, I71, I76, and E106 resides in SARS-CoV-2 ORF8 structure. (B) Binding activity of ORF8 mutants to THP-1 cells. THP-1 cells were incubated with purified ORF8 WT or mutants for 2 h, followed by flow cytometry with anti-Flag antibody. (C, D) Binding activity of ORF8 mutants to hIL-17RA (C) or hIL-17RC (D). At 24 h posttransfection with hIL-17RA (C) or hIL-17RC (D) expression plasmids, 293T cells were subsequently incubated for 30 min with mock treatment or purified ORF8 WT or mutants, followed by flow cytometry with anti-Flag antibody. (E) Signaling activity of ORF8 mutant to induce inflammatory cytokine gene expressions. THP-1 cells were mock treated or treated with ORF8 WT or mutants for 16 h, followed by RT-qPCR to measure the mRNA levels of the indicated genes, with horizontal lines indicating median values of the mRNA levels of the indicated genes in mock-treated THP1 cells observed. (F) PLA of ORF8 mutant-mediated hIL-17RA/C heterodimerization in NIH 3T3 cells. At 24 h posttransfection with HA-hIL-17RA and Myc-hIL-17RC, NIH 3T3 cells were treated with hIL-17A or purified ORF8 WT or mutants for 30 min, followed by PLA. *P* values were calculated by one-way ANOVA with Dunnett’s posttest in panels E and F. *, *P* < 0.05; **, *P* < 0.01; ***, *P* < 0.005; ****, *P* < 0.0001.

To further investigate the hIL-17RA–ORF8 interaction, we generated 12 ORF8 point mutations without compromising the protein’s structural integrity based on the Rosetta point mutant scan application, a computational algorithm designed to predict a change in structural stability induced by single or double mutations (34). We identified four ORF8 mutants (Y42H, E106P, I71D and I76D) that showed markedly decreased binding to hIL-17R on THP1 cells (Fig. 5A and B). Intriguingly, when 293T cells expressing hIL-17RA or hIL-17RC were used in the binding assay, the Y42H and E106P mutants showed the complete loss of hIL-17RA binding activity but maintained hIL-17RC binding activity compared with the WT (Fig. 5C). Conversely, the I71D and I76D mutants showed similar hIL-17RA binding activity but drastically reduced hIL-17RC binding activity compared to the WT (Fig. 5D). Moreover, all ORF8 mutants showed little or no induction of *CCL10*, *CXCL1*, and *IL6* expression (Fig. 5E). Finally, PLA showed that compared to ORF8 WT, the Y42H, I71D, I76D, and E106P mutants induced marked reduction of hIL-17RA/C heterodimerization (Fig. 5F). These results suggest that Y42 and E106 and I71 and I76 are critical residues for the ORF8 interaction with hIL-17RA and hIL-17RC, respectively.

### ORF8 variants associated with attenuated inflammation phenotype.

**FIG 6:**
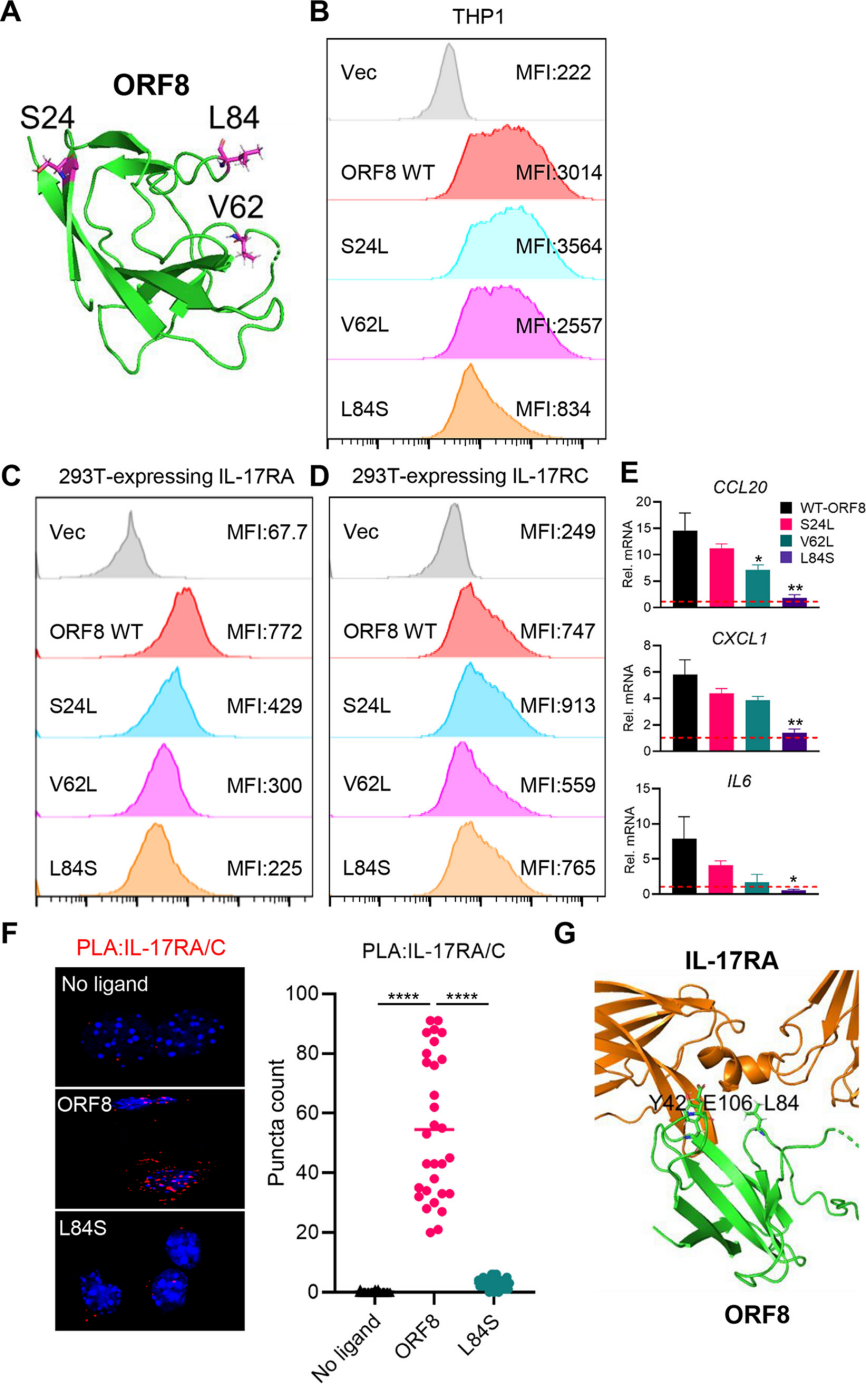
ORF8 natural variants for hIL-17R binding and inflammatory signaling activity. (A) S24, V62, and L84 residues in SARS-CoV-2 ORF8 structure. (B) Binding activity of ORF8 natural variants to THP-1 cells. THP-1 cells were incubated with mock treatment or ORF8 WT or variants for 2 h, followed by flow cytometry with anti-Flag antibody. (C, D) Binding activity of ORF8 natural variants to hIL-17RA (C) or hIL-17RC (D). At 24 h posttransfection with hIL-17RA (C) or hIL-17RC (D) expression plasmids, 293T cells were subsequently incubated for 30 min with mock treatment or purified ORF8 WT or variants, followed by flow cytometry with anti-Flag antibody. (E) Signaling activity of ORF8 variants to induce inflammatory cytokine gene expressions. THP-1 cells were mock treated or treated with ORF8 WT or variants for 16 h, followed by RT-qPCR to measure the mRNA levels of indicated genes, with horizontal lines indicating median values of the mRNA levels of indicated genes in mock-treated THP1 cells observed. (F) L84S variant shows the loss of IL-17RA/RC heterodimerization. At 24 h posttransfection with HA-hIL-17RA and Myc-hIL-17RC, NIH 3T3 cells were treated with the ORF8 WT or L84S variant for 30 min, followed by PLA. (G) Predicted structural representation of the Y42, Y106, and L84 residues at the binding interface between ORF8 and IL-17RA. *P* values were calculated by one-way ANOVA with Dunnett’s posttest in panels E and F. *, *P* < 0.05; **, *P* < 0.01; ***, *P* < 0.005; ****, *P* < 0.0001.

Recent studies have identified ORF8 as a hot spot of SARS-CoV-2 variations, and patients infected with SARS-CoV-2 carrying ORF8 variants often show mild disease symptoms (23, 35, 36). Three variants, S24L, V62L, and L84S, the most frequently emerged ORF8 variants in America (36, 37) (Fig. 6A), were selected to test their hIL-17R interaction and downstream inflammatory activation. When hIL-17RA-expressing or hIL-17RC-expressing 293T cells and THP-1 cells were incubated with purified the ORF8 WT or one of the L84S, V62L, and S24L variants, the L84S variant displayed the most drastic reduction of the surface binding activity to THP-1 cells and hIL-17RA-expressing 293T cells but not hIL-17RC-expressing 293T cells compared with WT ORF8 (Fig. 6B to D). The S24L and V62L variants also showed detectable reduction of the surface binding activity to THP-1 and hIL-17RA-expressing 293T cells but minor changes of the surface binding activity to hIL-17RC-expressing 293T cells (Fig. 6B to D). Correspondingly, the L84S variant failed to induce *CCL20*, *CXCL1*, and *IL6* expression, whereas the V62L and S24L variants induced detectably lower expression of *CCL20*, *CXCL1*, and *IL6* compared to WT ORF8 (Fig. 6E). PLA showed that the L84S variant, which is predicted to be located near the Y42 and E106 residues at the ORF8–IL-17RA binding interface, also induced dramatically reduced hIL-17RA/C heterodimerization compared to WT ORF8 (Fig. 6F and G). These results demonstrate that the L84S change, among the most frequently emerged ORF8 variations, leads to reduced hIL-17RA binding activity and thereby attenuated inflammation, suggesting a potential mechanistic explanation of mild COVID-19 symptoms of patients infected with the ORF8 L84S variant.

## DISCUSSION

Growing evidence has indicated the association of the hyperactivated immune system with poor COVID-19 prognosis (38). Several studies have shown that structural proteins of SARS-CoV-2 induce excessive inflammatory responses. For instance, the SARS-CoV-2 envelope protein has been shown to activate the Toll-like receptor 2-mediated NF-κB pathway, while its nucleocapsid protein promotes NLRP3 inflammasome activation (39, 40). On the other hand, secreted viral proteins are often associated with virulence and immunomodulatory properties. For example, the secreted forms of the nonstructural protein 1 (NS1) of human norovirus and the negative regulatory factor (Nef) of human immunodeficiency virus (HIV) can regulate systemic immune responses, including in uninfected cells (41, 42). We and other groups identified the SARS-CoV-2 ORF8 as a secreted protein, whose abundant expression and antibody were readily detected in the lung tissues of SARS-CoV-2-infected animals and the sera of COVID-19 patients, respectively. Recent SARS-CoV-2 versus host protein interactome studies have identified the ORF8 interaction with hIL-17RA, a crucial receptor in IL-17 signaling (30, 31). Indeed, the pronounced activation of inflammatory IL-17 signaling has been reported in COVID-19 patients (9). Intriguingly, Gamage et al. found that while SARS-CoV-2 primarily affects the lung, SARS-CoV-2 infection of airway epithelial cells leads to a restricted cytokine release profile, thereby making a contribution to the cytokine storm detected in the peripheral blood of COVID-19 patients unlikely (43). Herein, we showed that treatment of whole blood with purified ORF8 led to the robust activation of inflammatory responses, including NF-κB-driven cytokine genes—*IL6*, *CXCL1*, *CXCL2*, and *CCL20*—and the upregulation of hIL-17R signaling. In addition, transcriptomic analysis suggested that ORF8 exclusively upregulated the expression of key factors involved in fibrosis signaling and coagulation dysregulation, the common clinical manifestations of COVID-19 patients (44, 45). Our study uncovers SARS-CoV-2 ORF8 as a viral cytokine that stimulates peripheral immune cells to induce hIL-17R-mediated inflammation.

Blood monocytes are the source of hyperinflammatory cytokine production during SARS-CoV-2 infection (46). We identified human monocytes as the primary immune target cells where secreted ORF8 interacted with hIL-17R on the surface and induced the production of proinflammatory factors. While hIL-17RA is ubiquitously expressed on many cell types, ORF8 interacted primarily with monocytes, suggesting that additional host factors may be involved for ORF8 interaction with monocytes. It should be also noted that our finding of ORF8-mediated induction of hIL-17R signaling in monocytes is consistent with a recent study of Lin et al. (33), albeit using murine RAW264.7 monocytic cells. In contrast to these findings, we showed the specific interaction of ORF8 with human primary monocytes and human THP-1 and U937 monocytic cells, but we were unable to show ORF8 interaction with mouse RAW264.7 cells. We also showed the specific interaction of ORF8 with hIL-17RA and hIL-17RC, but not mIL-17RA, whereas mIL-17 was readily bound to mIL-17R and induced downstream signaling under the same conditions. A possible explanation for this discrepancy may be the difference in the source of ORF8. Due to its secreted and glycosylated nature, ORF8 was purified from the supernatants of transfected 293T cells in our study, whereas Lin et al. (33) used bacterially purified ORF8. In addition, it is unclear whether bacterially purified ORF8 was free of endotoxin for stimulation since no information on that was given in the study (33). Regardless, our study clearly demonstrates that SARS-CoV-2 ORF8 interacts with hIL-17R on human monocytes and induces the IL-17R signaling pathway.

SARS-CoV-2 is an RNA virus that is prone to a high rate of mutation. In fact, 5 out of the 29 SARS-CoV-2 proteins—nucleocapsid, ORF3a, ORF8, RNA-dependent RNA polymerase, and spike—have been identified as major hot spots for mutations (47, 48). Clinical studies have shown that COVID-19 patients infected with naturally occurring ORF8 deletion or ORF8-L84S variant virus had mild symptoms, suggesting ORF8 as a potential virulence factor that contributes to COVID-19 disease severity (22, 23). To understand the mechanism underlying the reduced virulence of ORF8 variants, we tested the three most predominant ORF8 variants (28). While all three variants exhibited reduced binding activity to hIL-17RA, the L84S variant demonstrated the loss of hIL-17RA binding activity as well as the lack of proinflammatory cytokine production activity. This provides insight into the underlying mechanism of mild clinical outcomes associated with ORF8 variant virus infection.

IL-17RA is a common subunit shared among 5 different homo-/heterodimeric receptor complexes that induce IL-17 signaling. Among these receptor complexes, the IL-17RA/C heterodimeric complex serves as an obligate receptor for binding to IL-17A, IL-17F, and IL-17A/F, enabling activation of IL-17 signaling (11). We found that ORF8 specifically interacted with hIL-17RA and hIL-17RC and that these interactions induced the heterodimerization of hIL-17RA/C to activate pronounced downstream inflammatory responses, whereas depletion of the *IL17RA* or the *IL17RC* suppressed ORF8-mediated inflammatory response. Unlike the ORF8 WT, the naturally occurring ORF8 L84S variant induced neither the heterodimerization of hIL-17RA/C nor the production of proinflammatory factors. Computational modeling predicts that the L84 residue is located near the Y42 and E106 residues at the IL-17RA–ORF8 binding interface. In fact, the L84S variant was unable to induce hIL-17RA/C heterodimerization like the Y42H and E106P mutants. It is also intriguing that the Y42H and E106P mutants showed next to no hIL-17RA binding activity but maintained hIL-17RC binding activity, whereas the I71D and I76D mutants showed similar hIL-17RA binding activity but drastically decreased hIL-17RC binding activity compared to the WT. All ORF8 mutants failed to induce hIL-17RA/C heterodimerization and *CCL10*, *CXCL1*, and *IL6* expression. These findings suggest that the Y42- and E106-exposed side and the I71- and I76-exposed side of ORF8 dimer may independently interact with hIL-17RA and hIL-17RC, respectively, which leads to the robust heterodimerization of hIL-17RA/C and, thereby, the pronounced activation of downstream inflammatory signal transduction. Future studies directed to test this hypothesis are ongoing.

In summary, we identified SARS-CoV-2 ORF8 as a viral mimic of the hIL-17 cytokine that contributes to COVID-19 severe inflammation. Current circulating ORF8 variant strains are often associated with clinically reduced virulence. Being a hot spot for variations, however, there is always a possibility that a new ORF8 variant strain emerges that has acquired enhanced virulence. Hence, our findings suggest SARS-CoV-2 ORF8 as a potential pathogenic marker and therapeutic target for severe COVID-19.

## MATERIALS AND METHODS

### Viruses.

SARS-CoV-2 USA-WA1/2020 strains were purchased from BEI Resources (NR-52281). All SARS-CoV-2 virus stocks were propagated in the Vero cell line (ATCC CCL-81) stably expressed with human ACE2. Viral titers were determined by plaque assays on Vero-ACE2 cells. SARS-CoV-2 wild-type and ORF8-deleted bacmid constructs were kindly provided by Armin Ensser (Institute of Virology, Erlangen, Germany) (49).

### Human subjects and blood collection.

Healthy serum samples (*n = 9*) were collected before the COVID-19 pandemic. Prospective serum samples for COVID-19-positive hospitalized (*n =* 40) and ICU patients (*n =* 9) were acquired from the Cleveland clinic COVID-19 Biobank. All sample collections occurred within 5 days of hospital or ICU admission for COVID. COVID positivity was confirmed by reverse transcription-PCR (RT-PCR) test. Blood was collected in a serum-separating tube, centrifuged at 2,000 × *g* for 10 min, and immediately aliquoted and stored at −80°C. All samples were processed in biosafety level 2 (BSL2)-enhanced facilities. All participants provided written informed consent before initiation of study procedures. The COVID-19 Biobank was approved by the Cleveland Clinic Institutional Review Board and Internal Biosafety Committee. Peripheral blood specimens for flow cytometry and transcriptome sequencing (RNA-Seq) were obtained from healthy donors aged 21 to 50 years old and stored temporarily at 37°C in lithium heparin-containing tubes (BD) prior to treatment. Blood specimens used in the figures were obtained from male and female subjects.

### Enzyme-linked immunosorbent assay.

Mouse monoclonal anti-ORF8 antibody was purchased from R&D (MAB10820) and used as the capture antibody in the capture ELISA. Rabbit polyclonal anti-ORF8 antibody was purchased from GeneTex (GTX135591) and biotinylated using the Biotinylation kit/Biotin Conjugation kit (Fast, Type A)-Lightning-Link (ab201795; Abcam) and used as the detection antibody. A 96-well ELISA plate was coated with 1 μg/mL monoclonal anti-ORF8 antibody diluted in coating buffer and incubated in a cold room for 16 h. Unbound antibodies were washed and removed, and 100 μL of blocking solution (phosphate-buffered saline with 0.05% Tween 20 [PBST] and 3% bovine serum albumin [BSA]) was added per well. The plate was then incubated for an hour at room temperature. Serum samples were diluted in 3% BSA–PBST at 1:5 and 1:50 and added (100 μL/well) to each well, and the plates were incubated for 90 min at room temperature. Biotinylated polyclonal anti-ORF8 antibodies were added at a concentration of 1 μg/mL. The plates were incubated for 1 h at room temperature and then washed 4 times with PBST. A 100-μL/well 1:10,000 dilution of streptavidin-horseradish peroxidase (HRP) in 3% BSA–PBST was added, and then the mixture was incubated for 1 h at room temperature. After a final washing 4 times, substrate was added (100 μL/well). The plates were incubated for 10 to 30 min, and then stop solution was added (100 μL/well). Luminescence was measured. Human CXCL1 (Invitrogen) and IL-6 (BD biosciences) ELISAs were performed on plasma specimens according to the manufacturer’s instructions.

### Plasmid constructs.

Genes encoding ORF7b and ORF8 were cloned into the pIRES-puro vector with a C-terminal 3×Flag. The pCMV6-IL-17RA and IL-17RB constructs were purchased from Origene. The IL-17RC plasmid was kindly provided by Pinghui Feng (University of Southern California). pCMV4Neo-murine IL-17RA-HA was a gift from Sarah Gaffen (50) (Addgene plasmid no. 46859; RRID:Addgene_46859 [http://n2t.net/addgene:46859]).

### Mammalian cell lines and primary cells.

HEK293T, RAW264.7, NIH 3T3, and Vero E6 cells were purchased from the American Type Culture Collection (ATCC) and maintained in Dulbecco’s modified Eagle’s medium (DMEM; Gibco) supplemented with 10% fetal bovine serum (FBS; Gibco) and 1% penicillin–streptomycin (Gibco). THP-1 and U937 cells were purchased from ATCC and maintained in RPMI 1640 medium supplemented with 10% FBS and 1% penicillin–streptomycin. Primary bone marrow differentiated macrophages were differentiated and cultured as described previously (51).

### Immunohistochemistry.

Tissue samples were collected from PBS control and SARS-CoV-2-infected ferrets or mice as previously described (52). Tissues were incubated in 10% neutral buffered formalin for fixation before they were embedded in paraffin based on standard procedures as described before. The embedded tissues were sectioned and dried for 3 days at room temperature. To detect the viral antigen by immunohistochemistry (IHC), rabbit polyclonal antibody of ORF8 purchased from GeneTex was used as the primary antibody. Antigen was visualized using the biotin-avidin system (Vector Labs). Slides were viewed using the Olympus IX 71 (Olympus, Tokyo, Japan) microscope with DP controller software to capture images (×400).

### Expression and purification of proteins.

HEK293T cells were transiently transfected with respective plasmids by using the calcium phosphate precipitation method. Supernatants containing ORF8 were harvested 72 to 96 h after transfection and concentrated with the Labscale TFF system equipped with filters (Millipore Sigma) for 3 kDa and 10 kDa. Cells containing ORF7b were harvested 32 h after transfection and lysed with lysis buffer. The concentrates (ORF8) or cell lysates (ORF7b) were incubated overnight with EZview Red anti-Flag M2 affinity gel (Sigma) at 4°C. The recombinant protein was eluted with 3×Flag peptide (Sigma). Purified protein was verified for their yield and purity via SDS-PAGE and stored at −80°C.

### *In vitro* whole-blood treatment and immune cell isolation.

Anticoagulated whole-blood specimens were incubated with purified Flag-tagged ORF8 protein at 37°C with intermittent shaking for indicated time courses. Isolation of immune cells or plasma was performed by density gradient centrifugation using lymphocyte separation medium (Corning). Plasma specimens were collected and stored at −80°C prior to use. Isolated immune cells were processed for further utilization.

### Flow cytometry.

Surface staining of indicated subsets was performed with the following antibodies: CD45-BV421 (clone HI30; Biolegend), CD14-Alexa Fluor 488 (AF488) (clone 63D3; Biolegend), CD56-peridinin chlorophyll protein (PerCP)-Cy5.5 (clone 5.1H11; Biolegend), CD3-AF647 (clone HIT3a; Biolegend), and CD19-PerCP (clone HIB19; Biolegend). ORF8-Flag staining was performed using phycoerythrin (PE)-conjugated fluorescence-activated cell sorter (FACS) antibody (Biolegend). FACS acquisitions were performed on BD FACSCanto II (BD), using BD FACSDIVA software. All FACS data were analyzed using FlowJo software.

### Immunoblotting analysis.

Cells were transfected or treated with the respective plasmids or indicated treatment. Whole-cell lysates were lysed with NP-40 lysis buffer. Samples were loaded on SDS-PAGE gels and transferred to a polyvinylidene difluoride (PVDF) membrane by semidry transfer (Trans-Blot Turbo; Bio-Rad Laboratories). All membranes were blocked in 5% milk in Tris-buffered saline with Tween 20 (TBST [pH 8.0]; Sigma-Aldrich) and probed overnight with the indicated antibodies. The primary antibodies included mouse anti-Flag (1:5,000 dilution, catalog no. B3111; Sigma-Aldrich), mouse anti-β-actin (1:5,000 dilution, catalog no. sc-47778; Santa Cruz Biotechnology), rabbit anti-ORF8 (1:500 dilution, catalog no. GTX135591; GeneTex), mouse anti-GAPDH (anti-glyceraldehyde-3-phosphate dehydrogenase) (1:5,000, catalog no. sc-47724; Santa Cruz Biotechnology), rabbit anti-IL-17RA (1:1,000 dilution, catalog no. 12661; Cell Signaling Technology), rabbit anti-IL-17RB (1:1,000 dilution, catalog no. 20673-1-AP; Proteintech), rabbit anti-IL-17RC (1:500 dilution, catalog no. 15648-1-AP; Proteintech), rabbit anti-phospho-NF-κB p65 (Ser536) (1:1,000 dilution, catalog no. 3033; Cell Signaling Technology), rabbit anti-phospho-IκBα (Ser32) (1:1,000 dilution, catalog no. 2859; Cell Signaling Technology), mouse anti-IκBα (1:1,000 dilution, catalog no. 4814; Cell Signaling Technology), and rabbit anti-NF-κB p65 antibody (1:3,000 dilution, catalog no. sc-8008; Santa Cruz Biotechnology). The appropriate horseradish peroxidase (HRP)-conjugated secondary antibodies—anti-rabbit IgG-HRP (1:2,000 dilution, catalog no. 7074S; Cell Signaling Technology) or anti-mouse IgG-HRP (1:2,000 dilution, catalog no. 7076V; Cell Signaling Technology)—were incubated on membranes in 5% milk in TBST at room temperature for 1 h. Protein bands were developed with HyGlo enhanced chemiluminescence reagent (Denville Scientific), imaged on ChemiDoc Touch (Bio-Rad Laboratories), and analyzed with Image Lab v.5.2.1 (Bio-Rad Laboratories).

### RNA extraction and RT-qPCR.

Total RNA extractions were performed using an RNeasy minikit or microkit (Qiagen) according to the manufacturers’ instructions. The RNA concentration was determined by NanoDrop 1000 spectrophotometer (Thermo Scientific). Extracted total RNA was reverse transcribed using iScript cDNA synthesis kit (Bio-Rad) according to the manufacturer’s instructions. SsoAdvanced Universal SYBR green supermix (Bio-Rad) was used for quantitative PCR (qPCR) according to the manufacturer’s instructions. Each sample was normalized to GAPDH, and the threshold cycle (ΔΔ*C_T_*) fold change method was used to calculate relative quantification. Primer sequences used for reverse transcription-qPCR (RT-qPCR) are found in Table S1.

10.1128/mbio.00402-22.5 TABLE S1 Primer sequences for qRT-PCR. Download Table S1, PDF file, 0.1 MB. Copyright © 2022 Wu et al. 2022 Wu et al. https://creativecommons.org/licenses/by/4.0/ This content is distributed under the terms of the Creative Commons Attribution 4.0 International license.

### Pan monocyte isolation and RNA sequencing.

Total monocyte populations were isolated from peripheral blood mononuclear cells (PBMCs) using a Pan monocyte isolation kit (Miltenyi Biotec), according to the manufacturer’s instructions. Total RNA was isolated with an RNeasy microkit (74004) from Qiagen, and the RNA-Seq library was prepared using the KAPA mRNA HyperPrep kit (KR1352), according to the manufacturers’ instructions. Sequence reads were obtained using Illumina NovaSeq 6000 as well as the Illumina MiSeq systems with the help of the Genomics Core in the Cleveland Clinic Foundation. Read counts were normalized on the basis of reads per kilobase per million mapped reads (RPKM), and fold changes were calculated for all possible comparisons. Genes with a fold change of >2 are considered to be induced significantly. The analysis of pathway and transcription factors regulated by ORF8 or IL-17A was performed by the Ingenuity Pathway Analysis (Qiagen). The data discussed in this publication have been deposited in NCBI's Gene Expression Omnibus (53).

### CRISPR-Cas9 knockout.

The protocols for genome engineering using the CRISPR-Cas9 system were previously described (54). Briefly, double-stranded oligonucleotides corresponding to the target sequences were cloned into the lenti-CRISPR-V2 vector and cotransfected with packaging plasmids into HEK293T cells. Two days after transfection, the viruses were harvested and used to infect the indicated cells. The infected cells were selected with puromycin (1 μg/mL) for at least 5 days. The following sequences were used: human IL-17RA cDNA, 5′-CACCGGTGGTCCAGGAGTCGCAGGG-3′; human IL-17RB cDNA, 5′-CACCGGTCGCTCGTGCTGCTAAGCC-3′; and human IL-17RC cDNA, 5′-CACCGCCGGTGAGTCTGGAACCCTG-3′.

### Modeling of ORF8–IL-17RA interaction.

The crystal structures of ORF8 (PDB ID 7JTL) and IL-17RA (PDB ID 5N9B) were retrieved from Uniprot (37). The Rosetta software was used to simulate the structural model of SARS-CoV-2 ORF8 with IL-17RA (55, 56). The Rosetta point mutant scan application was performed to predict the protein stability for ORF8 mutations (57). Global docking simulations were performed by RosettaDock 4.0 using RosettaScripts with refinement and FastRelax algorithms (58, 59). The 10,000 decoys were ranked by their total score: the best model was chosen by the experimental results (Fig. 5), which allows us to narrow down the binding interface by the positions of key residues (Y42, E106, I71, and I76).

### *In situ* proximity ligation assay.

NIH 3T3 cells were transfected with HA-tagged IL-17RA and Myc-IL-17RC for 24h. Cells were collected and immediately fixed in 4% paraformaldehyde (PFA) for 15 min and thereafter subjected to *in situ* PLA using the Duolink In Situ Detection Reagents Orange kit (Sigma-Aldrich), according to the manufacturer's instructions. Different combinations of primary antibodies to HA and Myc were used. Then, PLA signals were detected by a Keyence confocal laser scanning microscope using a 60× oil immersion objective. High-resolution images (taken using a 60× objective) from each of the single scans were analyzed by ImageJ (NIH) to calculate the numbers of PLA puncta.

### Statistical analyses.

All statistical analyses were performed using GraphPad Prism 9.0 software. For analyses between 2 groups, multiple *t* tests were used. For comparisons among more than 2 groups, either one-way or two-way analysis of variance (ANOVA) with Tukey’s test, Dunnett’s test, or Bonferroni’s test was used.

### Data availability.

The data supporting the findings of this study are available in the article and supplemental material. All primers and probe sequences used in this study are available upon request. The data deposited in the NCBI Gene Expression Omnibus (53) are accessible through GEO series accession no. GSE196455.
