# Supplementary figures and images for: Viral Mimicry of Interleukin-17A by SARS-CoV-2 ORF8
Source: mBio. 2022 Mar 28;13(2):e00402-22. doi: 10.1128/mbio.00402-22 (PMC9040823; doi:10.1128/mbio.00402-22)

Figure S1.

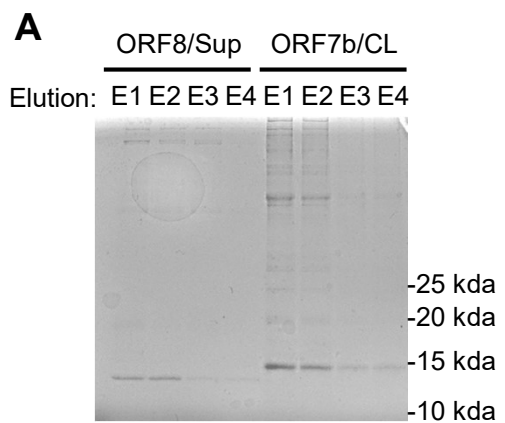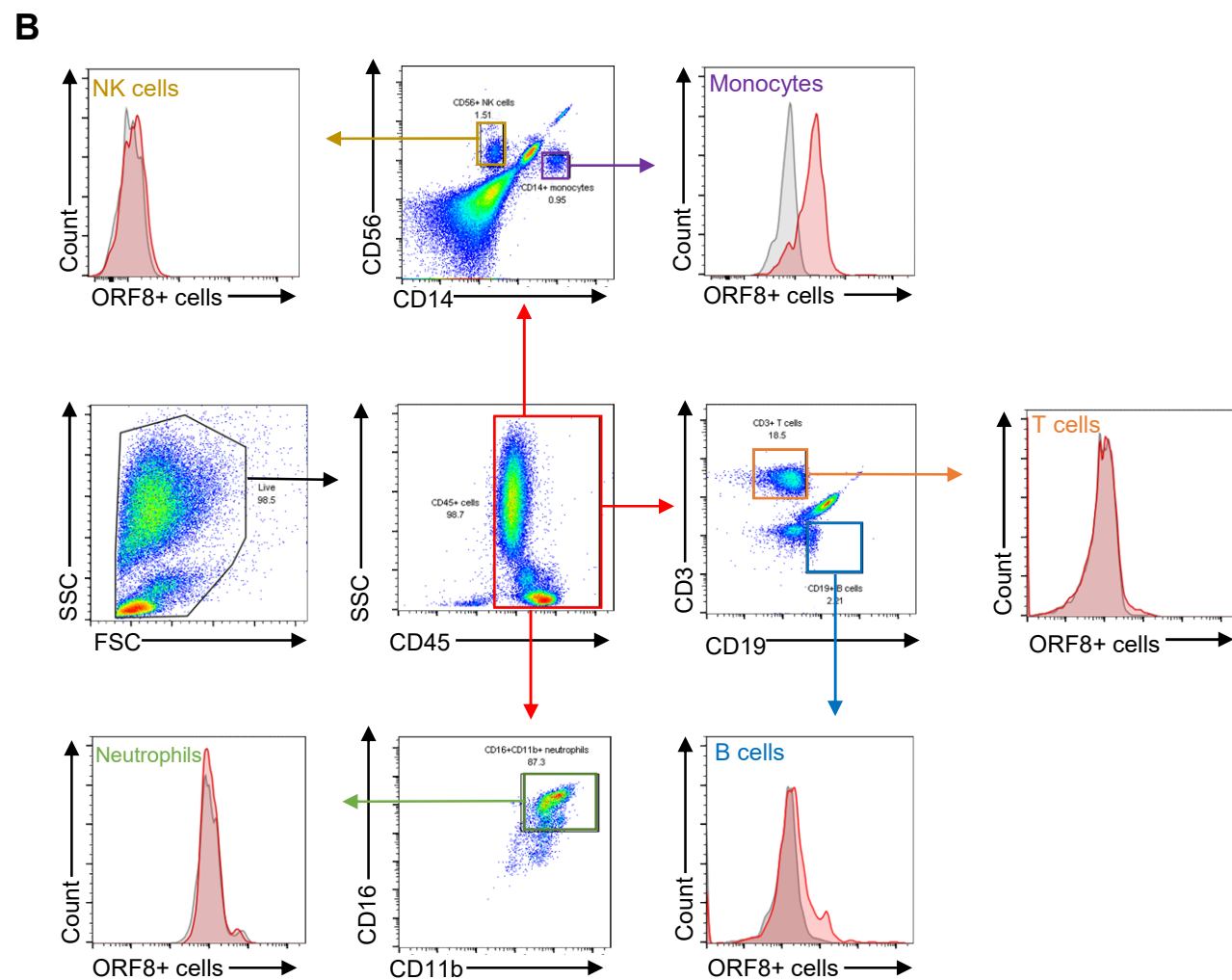

Supplement: FIG S1 [file mbio.00402-22-sf001.pdf]

Figure S2.

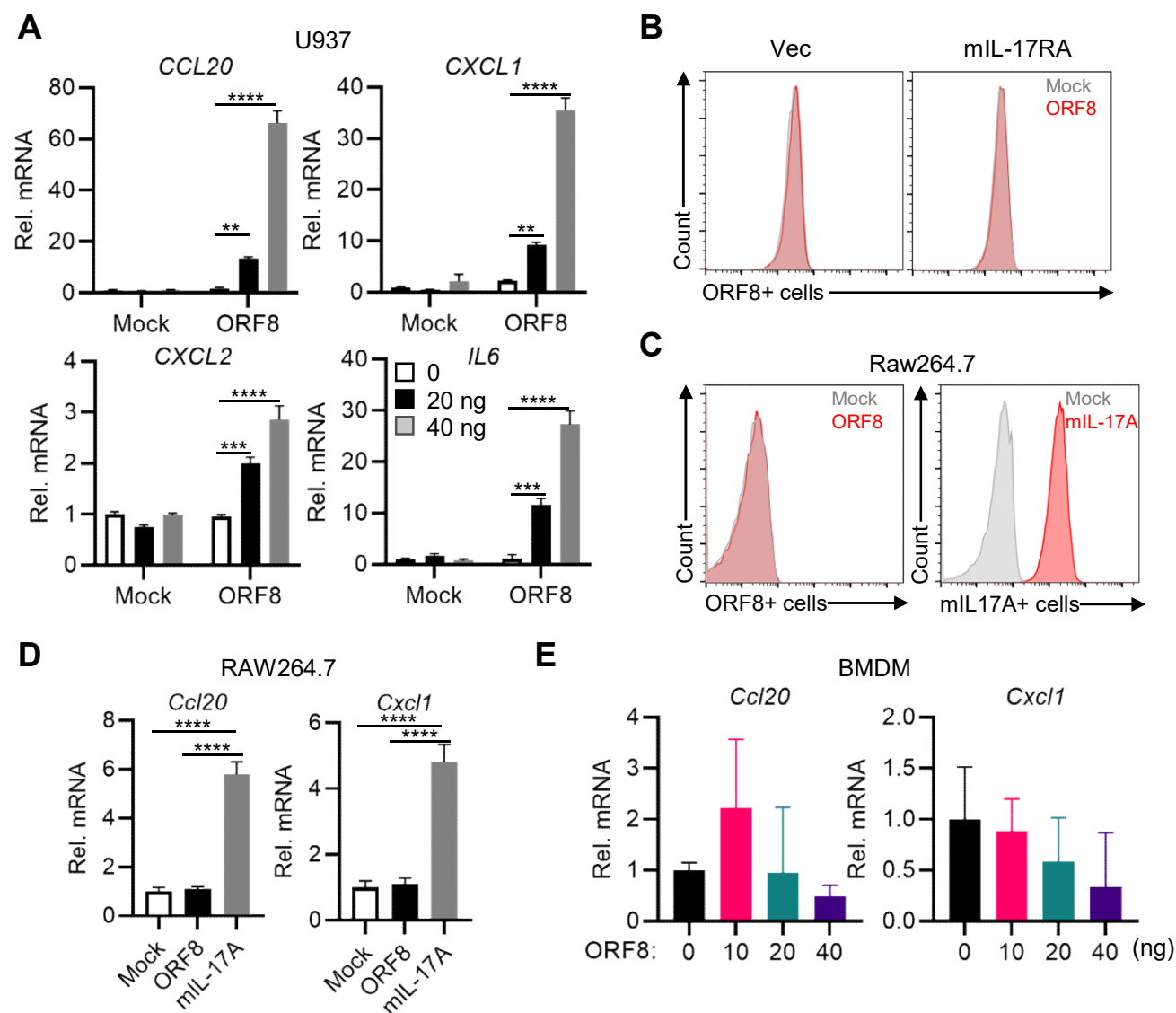

Supplement: FIG S2 [file mbio.00402-22-sf002.pdf]

Figure S3.

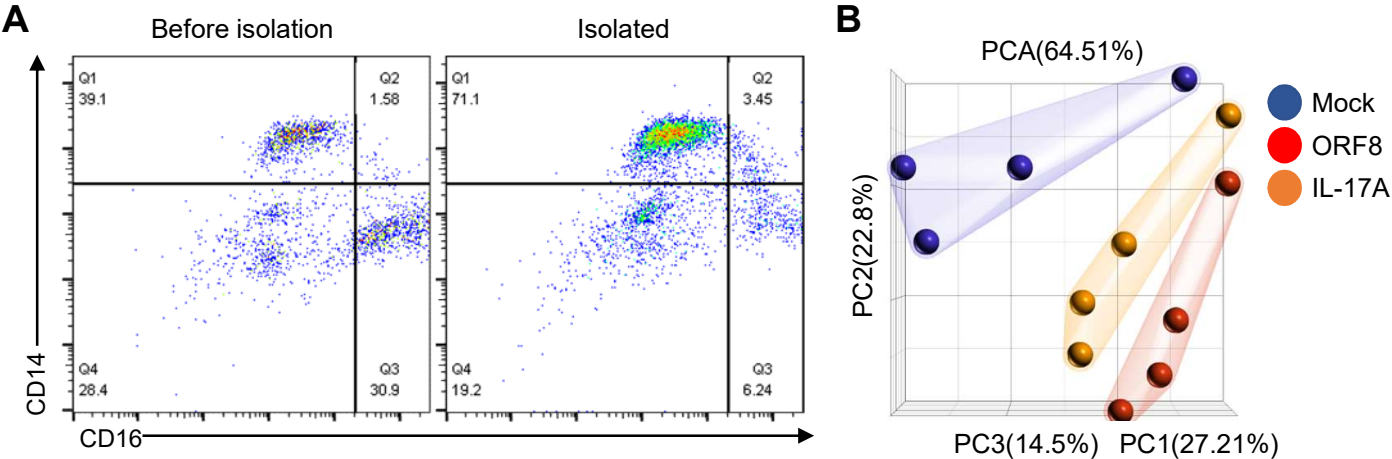

Supplement: FIG S3 [file mbio.00402-22-sf003.pdf]

Figure S4.

**A**

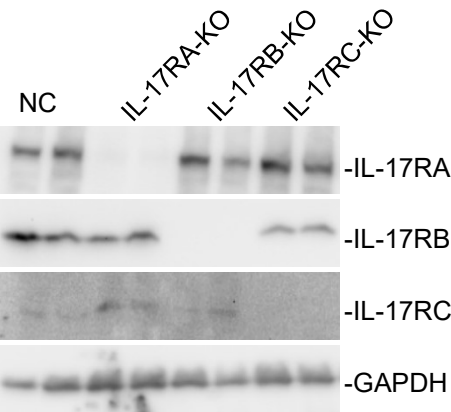

**B**

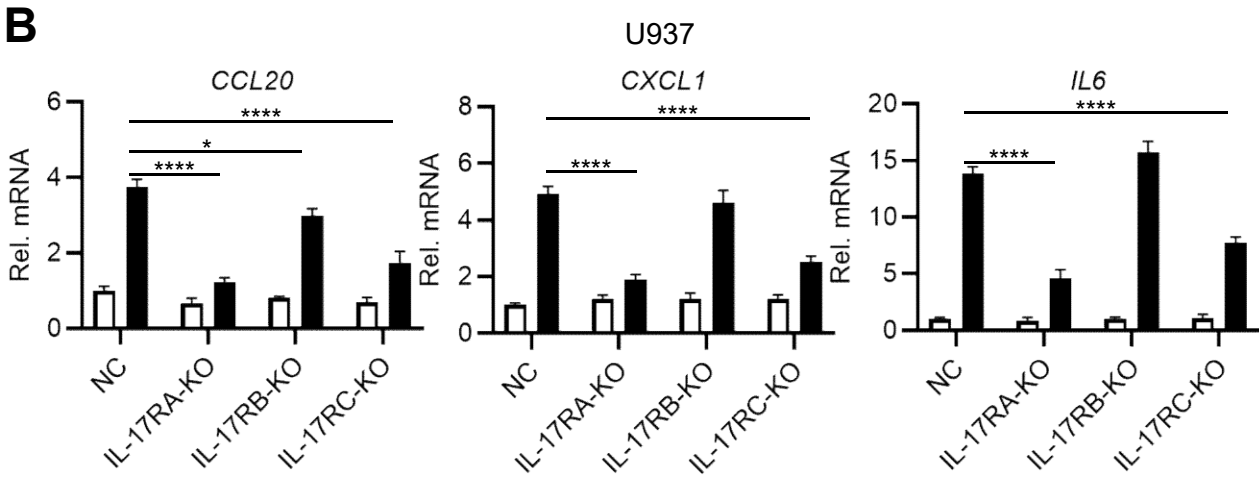

Supplement: FIG S4 [file mbio.00402-22-sf004.pdf]
