# Supplementary material for: Viral Mimicry of Interleukin-17A by SARS-CoV-2 ORF8
Source: mBio. 2022 Mar 28;13(2):e00402-22. doi: 10.1128/mbio.00402-22 (PMC9040823; doi:10.1128/mbio.00402-22)
Supplement: TABLE S1 [file mbio.00402-22-st001.pdf]

Table S1. qPCR primers

GAPDH-F	GACAAGCTTCCCGTTCTCAG
GAPDH-R	GAGTCAACGGATTTGGTCGT
CCL20-F	TGCTGTACCAAGAGTTTGCTC
CCL20-R	CGCACACAGACAACTTTTTCTTT
CXCL1-F	GAAAGCTTGCCTCAATCCTG
CXCL1-R	CTTCCTCCTCCCTTCTGGTC
CXCL2-F	GGGCAGAAAGCTTGTCTCAA
CXCL2-R	GCTTCCTCCTTCCTTCTGGT
IL-6-F	AGACAGCCACTCACCTCTTCAG
IL-6-R	TTCTGCCAGTGCCTCTTTGCTG
CSF3-F	TCCAGGAGAAGCTGGTGAGTGA
CSF3-R	CGCTATGGAGTTGGCTCAAGCA
IL12B-F	GACATTCTGCGTTCAGGTCCAG
IL12B-R	CATTTTTGCGGCAGATGACCGTG
COL17A1-F	GCTCTTGGCATTCCTAGTGGTC
COL17A1-R	GATGTACTGCTGAATCTCCTGGC
MMP10-F	TCCAGGCTGTATGAAGGAGAGG
MMP10-R	GGTAGGCATGAGCCAAACTGTG
SERPINB2-F	GCTGTTTGGTGAGAAGTCTGCG
SERPINB2-R	CTGCACATTCTAGGAAGTCTACTG
Gapdh-F	CATCACTGCCACCCAGAAGACTG
Gapdh-R	ATGCCAGTGAGCTTCCCGTTCAG
Cxcl1-F	AGACCATGGCTGGGATTCAC
Cxcl1-R	CAAGGGAGCTTCAGGGTCAA
Ccl20-F	CGACTGTTGCCTCTCGTACA
Ccl20-R	GAGGAGGTTCACAGCCCTTT
